# Tracing the journey of *Thattai Bhatia* community through their culinary identity

**DOI:** 10.1186/s42779-021-00108-1

**Published:** 2021-11-08

**Authors:** Navreet Kaur Rana

**Affiliations:** grid.449565.f O.P. Jindal Global University, Sonipat, India

**Keywords:** *Thattai Bhatia*, Culinary identity, Food history, *Pushtimarg*, *Sattvik*

## Abstract

The paper acknowledges the remarkable contribution of cookbooks which have always played an instrumental role in researching the history of any community. However, it brings to light the fact that there are several reasons like migration, small size of the community or the nomadic lifestyles when the culinary regime of the community could not be documented. In such cases, the everyday food choices of an ethnic community can lead us to tracing its origin and journey. The paper, thus, argues that in situations where there is paucity of literature documenting the culinary system or foodways, culinary identity of the community can become an effective method to trace the history of the community. The same is proved with the help of a case study of the *Thattai Bhatia* community. *Thattai Bhatia* is a small diaspora largely settled in the Persian Gulf, originally migrated from Rajasthan in India and later from Thatta in Sindh, Pakistan. The research reveals the reasons behind their distinct foodways such as abstinence from consuming liquor, meat, garlic and onion in particular, despite their intermingling with different ethnicities due to migration. The paper draws evidences from their regular foodways and traverses backwards to trace their origins, their history and the reasons that have shaped their contemporary food choices. With limited availability of literature, the author had to depend on the information provided during interviews by some of the community members about their food practices. All the findings are substantiated with references from the historical literature available.

## Introduction

History, in general, remains an endeavor to search the truth of oblivion of past in contemporary light. It helps to find newer avenues by excavating the treasure trove of a chronicled past. This also holds true for history of food, a discipline of scholarly interest for the last few decades. A surge in the number of monographs, cookbooks, infotainment and documentary format of food history programs has been noted recently. The roots of recording food history are nearly as old as writing history itself [1]. In the western tradition, Athenaeus of Naucratis documented in detail, ancient food habits in *Deipnosophistae* in the second century AD. Contemporarily, in the east, Meng Shen of T’ang dynasty wrote records of food consumed in court. In the South, Indian mythology records that the oldest and first ever book on cookery is *Pakadarpanam (*meaning *The Mirror of Culinary Skills*), written by the King Nala^1^ of the Nishada^2^ Kingdom. However, modern academic studies are estimated to have evolved around sixteenth century with Janus Cornarius’s *De conviviis veterum Graecorum* (1548) and J. Guglielmus Stuckius’s *Antiquitatum Convivialium* (1583) following which, food history began to exist as an academic subfield.

Regardless of the timeline, culinary jottings and cookbooks have always played an instrumental role in establishing cooking as a research methodology to study food-history [2]. Scholars in the past also believed that cookbooks have been a source of information not only to understand the domestic, social, or gender specific roles but also material factors such as trade and network [3]. Appadurai has considered cookbooks as humble literature of complex civilizations that publicizes particular traditions guiding the journey of food [4]. In the same article, he also pointed out an important fact that cookbooks seem to have come from royal and aristocratic milieus as they were the ones who could afford complex cuisines and had resources to record and document the cuisine [4]. The “resources” he mentions here need further attention as they may not necessarily be restricted to financial facets. For the subaltern, the resource-worthy reasons which could be responsible for the absence of documented cuisine of common people could be non-existence of a functional writing system, migration—both circumstantial and trade related, small size of the community, priority to sustain themselves, nomadic nature of their profession and geographical conditions among others.

In this paper, the question under research is that—in cases where there is complete absence or paucity of documentation of any culinary regime due to one or several reasons stated above, can the contemporary culinary identity of the community becomes that method which can lead to untangling the threads of historical events? The study will establish that a backpedaled journey from the food on the plate to the origin can help define the culinary praxis of any community and can become a method to study history of the community. An apt example of one such community is the *Thattai Bhatia* Community. The details and the reasons to choose this community as a case study here are discussed in the next section.

## The *Thattai Bhatia* community

The community derives its name from a place where one of the most prosperous civilization once flourished, Thatta, on the banks of river Indus. Thatta, (located at 24° 45′ N and 67° 58′ E) means “river bank”^3^ was once a flourishing town but gradually declined by the end of nineteenth century and today exists as a dusty provincial backwater [5].

The literature available on this community majorly addresses their trade relations and considers them as merchant diaspora [6] but is largely silent on their lifestyle. One more reason why the community could not mark a distinct presence was because of their small size. (No proper census is done on the population of the community. As per the web portal Bhatia.org, the population is estimated to be 5000.) They were overshadowed with either the Sindhi Hindu Community who also lived in Thatta and adjoining regions during the same period or with *Lohana*^4^ and *Arora*^5^ communities owing to the similarity in the nature of their profession. This fact is clearly visible in the works of Curtin [7], Dale [8] and Lala [9]. In the scholarly works of Curtin and Dale and the travelogue of Lala, the Sindhis are either referred to as merchants and *Lohanas* or *baniyas*.^6^ In fact, Schaflechner in his book about a Hindu temple (*Hinglaj Devi*) in the present-day Baluchistan clearly discusses the “absorption” of many other groups and clans in the *Lohana* community [10]. He remarks that “Over the centuries, the *Lohana* community absorbed many other castes from the western part of the subcontinent.”

It thus becomes difficult to isolate the cultural details of a smaller community in the recorded works. This essentially becomes another reason to choose their food and foodways to explore the history as Julia Darnton has stated that foodways are the windows into the culture and history of those who came before us [11].

## Theoretical background

The process of tracing the journey of a contemporary cuisine to its origin runs parallel to distinguishing a commutative relationship between food habits and community or region. This commutativity in relationship is achieved through identification. The food habits identify the community and this relationship exists vice-versa. However, the notion of associating food with identity is not new. Mintz and Du Bois [12], in their extensive work on the anthropology of food and eating, have acknowledged “Eating and Identities” as one of the seven topics that illuminate symbolic value-creation and the social construction of memory. They state that “Like all culturally defined material substances used in the creation and maintenance of social relationships, food serves both to solidify group membership to set groups apart”. Just like the constituent elements of food make an impact on our physique, our constituent food habits make us recognizable in our cultural system, thus becoming our identity.

What is there on our plate on a regular day, is nothing but a symbolic representation of our food choices. Now, if there is a pattern in the symbolic representation of the food, it tends to become the identity of practitioner. The more concrete the patterns are, stronger is the association of the identity. Just as Geertz [13] considered culture as “historically transmitted pattern of meanings embodied in symbols”, our cuisine has also travelled to us historically transmitting pattern of meaning embodied as dishes.

Scholars in the past have done remarkable work in establishing a relationship between the foods people eat and how others perceive them and how they see themselves [14, 15]. Anthropologists following Claude Lévi-Strauss [16], Jack Goody [17] or Mary Douglas [18], and sociologists following Pierre Bourdieu [19], rightly stress how consumption decisions express the civilized state, establish personal and collective identity, and mark cultural and social differences.

Whether it is the judgement of taste, as Bourdieu [19] stated identifies a social order or the Culinary Triangle of Strauss, which emphasizes that culinary habits are interpretable, they both lead to identification of some sort. On the other hand, Mary Douglus [18] in her work “Deciphering a Meal” partly disapproves of the Culinary Triangle but still continues to decipher a meal which means for her, food choices carry a meaning. She writes “If food is treated as a code, the messages it encodes will be found in the pattern of social relations being expressed”. There is enough scholarly literature on the establishment of the relationship of food and identity. The same identification also exists for the Thattai Bhatia community. They are recognized by their *Sattvik* food habits devoid of meat, alcohol, and onion. But the history behind the food choices remained in shadows. What has shaped their food habits to this? Or have they always been following these food choices? Why are their ethnic dishes similar to both Sindhi community and Rajasthan and Gujarat in India despite the fact they do not reside there? The rest of the paper indulges in finding an answer to these questions.

## Research methodology

This research is a retrospective exploration of the causes, the effect of which have already occurred. The research will explore the history of the *Thattai Bhatia* community with the help of their food choices and establish that in the absence the documentation of culinary regime, culinary identity becomes the resource for historical exploration. The population sample under review is the contemporary members of the community who mostly reside in modern day Oman, Bahrain, Dubai, Muscat and Mumbai. Given the inadequacy of relevant literature, the author had to depend upon the in-depth interview of the community members.

The guiding questions behind the choice of informants were two—first that they belong to the Thattai Bhatia community and second that they are informed, cook or are involved in practicing their cuisine irrespective of their age and gender. The informants were limited in number because of the small size of the community, their presence on social media and their knowledge and interest in cooking. The members who whose responses are captured in this paper are referred to as informants henceforth. Since the informants were largely based out of another country, I first contacted them through social media and then exchanged several messages knowing about their cuisine.^7^ Initially, the interviews of small number (8) of participants were not leading to conclusive outcomes but when all the informants who responded directed me to a local book which one of the community members had written, the research began to converge at a point. They also informed me that there is a website,^8^ which is an adaptation of the book titled *Panja Khada* (meaning Our Food in Sindhi language). I, then contacted the author of the book, Mr. Bharat Chachara [20], since one of my other informants had referred to him as the “pioneer of the *Thattai Bhatia* community in Dubai”. He shared many distinct insights on their cuisine and foodways in an in-depth interview, which became the guiding light during the course of the research. The interview comprised of open-ended questions and was not based on any assumptions. The interviews were conducted in the first quarter of 2021.

## Results

The key findings revealed that despite the fact that several features of the cuisine derive influence from the events in the history, the cuisine is actively practiced in Thattai Bhatia households every day. On asking that “if this is really the cuisine still practiced amongst the community members”, one of the informants responded affirmatively and said “Yes, very much. Cooked every day in all Bhatia household”. Another finding was the size of the population which the informant revealed that and “it is not more than 10,000 worldwide, however no formal counting or census has been conducted till date.” Regarding the literature available, he informed me that the physical copy of the book is available in Dubai and Mumbai only, but a digital copy is available on the website panjakhada.com and that it is a limited-edition book available only for private circulation. The informant also told me about a YouTube channel called “Buzzing Recipes” [21] run by them that streams 3-min videos of the *Thattai Bhatia* recipes.

The informant revealed that the younger generations today are accepting other “international cuisines like Chinese, Italian and Mexican” but at the same time are fond and aware of the Bhatia cuisine. Some of the houses strictly practice the ritual of *Bhog Dharanu* (discussed later) in their houses every day and annually celebrate the festival of *Annakut* (discussed later).

The strength of this information lies in its source. The information is coming from those who practice this culture in their day to day life as a primary chore. Another factor that helped in converging the research is the uniformity in the responses. They all directed me to refer to PanjaKhada.com which became a deciding factor to interview its author. On the basis of the information procured in the interviews, from the book and the videos, I was able to understand the ingredients and recipes of the food that makes it to their plates which was further tracked back into the history of the community. All arguments in the paper are supported with the historical facts available in the literature.

In the following sections of the paper, the foodways of the community are discussed, their resemblance and dissimilarities from Sindhi and Indian (from Gujarat^9^ and Rajasthan^10^) cuisine and through food the historical references are explored which have led to the formation of *Thattai Bhatia* cuisine to what it is as of today. In the later sections, the contemporary food choices of the community members are discussed, followed by a conclusion.

## The cuisine of *Thattai Bhatia* community

The members of the community refer to their food as “*Panja Khada”* which in Sindhi language means “Our Food”. The cuisine uses Bengal gram flour called *besan* in generous quantities and many dishes revolve around it. Also, many recipes such as *muthia*,^11^*mohan thaal*,^12^*sev tamatey* curry,^13^*dhokra*^14^ and *churmo*^15^ show remarkable similarities with the food and terminology of Gujarat^16^ and Rajasthan in India. This led to the first step of enquiry as to—are the community members even distantly related to Gujarat or Rajasthan? Since they derive their name from a place (Thatta) from undivided India, the possibility of any connection could not be ruled out.

Tracing back the roots revealed that the community ingeniously belonged to Jaisalmer which is in modern day Rajasthan (India). They belonged to the “Bhatti” clan who migrated from Rajasthan to Sindh around fourteenth century. The presence of Bhatti clan is mentioned in A Gazetteer of The Territories under the Government of the East-India Company [22]. The record mentions that the Bhutneer or Bhutnair (later renamed as Bhatner and is now known as Hanumangarh district in Rajasthan) was formally the principal place of Bhattis and that the Bhattis were Rajputs who had migrated from Bhatner approximately six centuries ago. Another noteworthy fact stated in Vol II of the gazette about Bhattis is that “the religious strictness of the Bhatti Rajpoots is relaxed in consequence of their continual intercourse with the Mussalmans to the westward”. Similarly, Tod mentions how the Bhatti tribe was accustomed to smoking opium in *hookas (pipe)* and that “To ask a Bhatti for a whiff of his pipe would be deemed a direct insult” [23].

On the contrary, considering the *Panja Khada* of the community, the present culinary habits did not seem to be as “relaxed” as cited by Tod in the paragraph above. The everyday food of the community members is devoid of meat, eggs, onion, garlic and any intoxicating ingredients. They cook simple recipes with local vegetables (like eggplant, ridge-gourd and bitter gourd) mostly finished with chopped coriander leaves. Though tempering with cumin and high dose of asafetida in the form of *vaghar* is an essential procedure, the cuisine makes minimal or no use of whole spices. And to almost all the curries, a slurry of roasted Bengal gram is poured in. It could be done to add body to the curry in the absence of onion. This fact was later validated by one of my informants. He informed me that in the absence of egg or onion, a solution of Bengal gram (called *Channe jo atto*) and water is added to the curry as a thickening agent. This solution is known as Mayer.

**Fig. 1:**
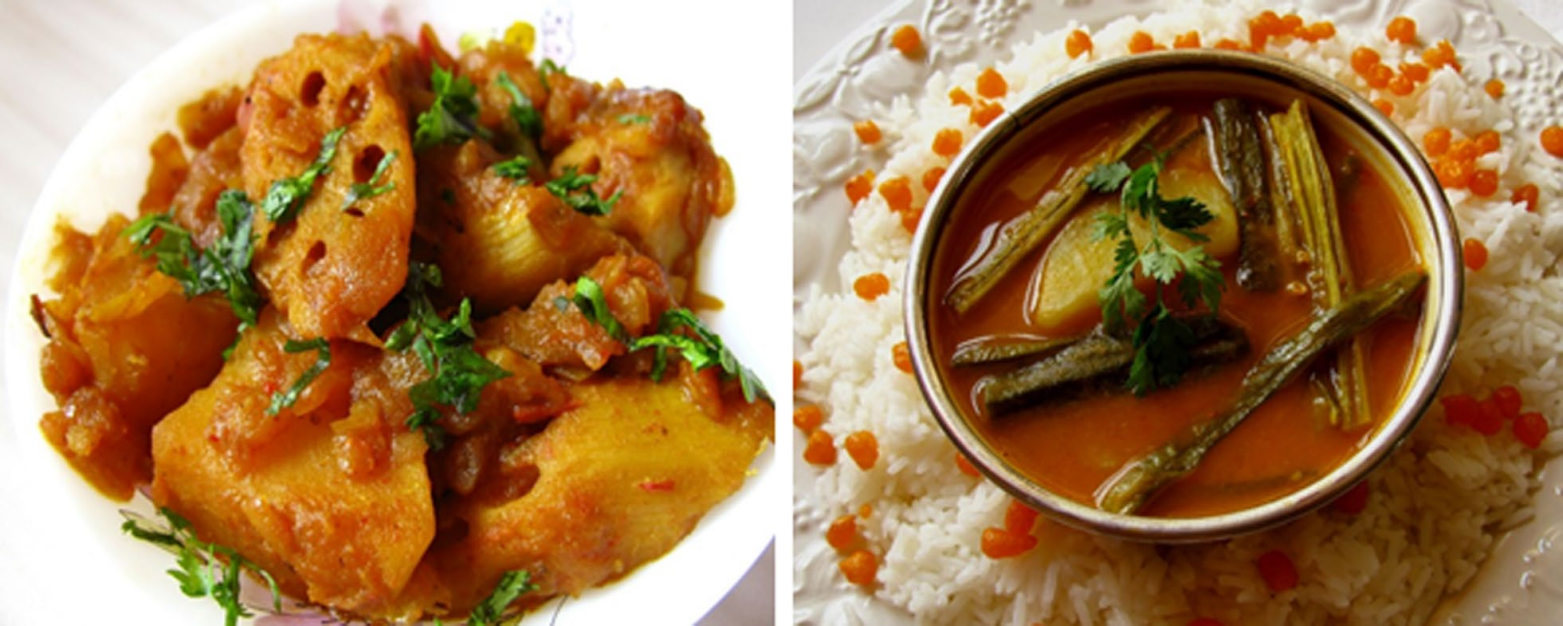
**a** Seyal bhey patata; **b** Sindhi kari. *Source*: Sindhi Rasoi: Sindhi Vegetarian & Vegan Recipes https://sindhirasoi.com/

The menu also shows striking similarities with that of Sindhi cuisine while maintaining the restrictions on onion, garlic and non-vegetarian products. The *Thattai Bhatia* cuisine shows extensive use of lotus-stem called *bhey* or *bhay* in the form of curries (*bhay gutter* & *bhay batate jo saag*^17^) (refer to image 1 (a)) and fritters (*bhay ja pakora*), which is a Sindhi favorite. Other preparations like a refreshing drink made out of jasmine flowers (*mogray jo sherbet), tuk,*^18^*kari*^19^*(Refer to* Fig. 1b),* dal pakwan*^20^ and *koki* (wheat bread usually had for breakfast) are quintessential to Sindhi cuisine. Despite adopting many Sindhi delicacies, the Bhatia cuisine has made a complete distance from *thoomro*^21^ (garlic), *pallo*^22^ (illish/hilsa/palla) and other fish and *macroli (*macaroni*)*.^23^ Also, these dishes are known by the same name in Sindhi cuisine.

The similarity of the *Thattai Bhatia* food with Gujarati-Rajasthani food is established by the presence of Bhatti clan in Jaisalmer. The similarity with Sindhi cuisine is indicative from the name of the community which they derived from Thatta, a town in Sindh. However, what let the Bhatti tribesmen move to Thatta and their abstinence from the use of non-vegetarian food, onion and garlic guides the research further.

Evidence shows that the Bhatti tribe from Jaisalmer (Rajasthan) migrated westwards to Thatta, a town in Sindh province [24]. This migration could be a circumstantial migration as the period of their migration overlaps with the period of decline of Rajputs around early fourteenth century after the Mughal invasion or it could be with an intent of trade. The tribesmen later established themselves as one of the oldest and strongest trading communities in Sindh who had strong commercial ties with the Portuguese and in the Gulf of Arabian Sea, especially Masqat (modern day Muscat) [24]. After late eighteenth century, they were clearly referred to as merchants as also classified by Scott Levi in his research on Indian diaspora as “merchant diaspora” [6]. Even so, a sudden transition from being a warrior clan to being traders doesn’t seem to be perceivably possible.^24^ A web portal^25^ on Bhatias mentions that in the early 1400’s, they were approached by the king of Thatta, who sought their help in putting down a rebellion in his kingdom. The Bhatias fought heroically and won back the king’s land. Obliged for their help, the king asked them to settle in Thatta itself. Yet, no historical evidence of this story is found in the literature.

Nonetheless, the *Bhatias* settled in Thatta and as mentioned in the beginning of the paper, it is from Thatta in Sindh, the community derived its name. Also, it is their stay in Thatta and neighboring regions, the striking similarity of their food with Sindhi cuisine is explained. However, their constraint from consuming animal products and onion and garlic remains unanswered.

The answer was found in the scholarly works of Richardson [25] and Burton [26] that the community members adopted *Vaishnavism,*^26^ a faith-inspired path of life. In the fifteenth century, Swami Vallabhacharya (1473–1531), a philosopher of the *Pushti* sect^27^ of *Vaishnavism* [27] visited Kutch, on the other side of Indus (on the banks of which lies Thatta). The devotees from Thatta crossed the river to hear the sermons and thereafter became the followers of *Pushtimarg*.^28^ The recognition of *Narayan Sarovar* (Refer to the map in Fig. 3) in Kutch on the other side of Indus, as one of the 84 seats (*baithaks*) of *Pushtimarg* tradition in India [25] ascertains the fact for the seats are sites where Vallabhacharya recited discourses from sacred scriptures.

Richard Burton in his work on the races in Sindh (1851) further writes that it was the followers of Vishnu who are forbidden to drink spirituous liquor or to eat meat, egg, fish, and onions [26]. He also writes that the priest of *bhatias* who worship Maharaja, an avatar of Vishnu is called *pokarno* and that he wears a *tilak* (mark on forehead) of horizontal lines distinguishing him as a *Vaishnav,* a practice followed by *Vaishnavs* throughout India [26].

The fact that *Thattai Bhatia* are followers of *Pushtimarg*, was also supported by the informants who were also kind enough to share certain other foodways of their community. They informed that that are a “traditional and conservative” community and not only abstain from consuming meat, fish poultry, onion and garlic, but also kidney beans and *masoor* (a kind of lentil). They follow a *Sattvik* (food with spiritual essence) diet and refrain from foods which induce *tamsik gun* (food which induces anger and laziness). Another reason they restrict themselves from eating meat and other products is that they offer their meals to the Lord (*Shrinath* or *Maharaj*, an avatar of Vishnu,) before consuming it. The food is then supposed to be blessed by the Lord and is consumed as a *Prasad* (blessed food). They call this ritual as *Bhog Dharanu*. As per one of my informants, many families still follow this ritual every day in their household. Some community members also observe a fast every month on the 11^th^ day of the lunar calendar which they call as *Igyas*. The person fasting consumes only one meal in a day which is devoid of grains and legumes. Apart from fasting, they also observe a ritual of feasting which is called *Annakut* (meaning mountain of food) and prepare a meal of 56 *sattvik* dishes. The meal is called *chhappanbhog* (*Chhapan* means numeral 56 and *bhog* means offering) and is offered on the day of *Goverdhan Puja.* Goverdhan is the name of the mountain in Mathura, (northern India) which Lord Krishna (an avatar of Lord Vishnu) lifted on his little finger when he was just 7 years old and held it for 7 days to protect the natives from incessant rains. To pay their homage to the lord, the villagers prepared 56 food items considering offering 8 items per day for 7 days. Toomey [28] has described, how significant the festival of *Annakut* is for *Vaishnav* followers and how it is celebrated across various places.

With these foodways in sight, it can be supposed that the faith of the community in *Pushtimarg* played a crucial role in shaping their cuisine. The cuisine is still meticulously practiced by the community despite their small size and having migrated from their indigenous homeland.

Most of the *Thattai Bhatia*s today live in the Gulf countries, mainly Muscat and Bahrain. With the decline of the port city of Thatta, Bhatias started moving to the Gulf through waterways. They had established intense commercial relations at Muscat by then [24]. Several accounts of the decline of Thatta and Bhatia traders in Muscat are available in literature. Although, the first mention of *banias* of Sind occurred in Arab and Portuguese documents concerning Masqat at the end of the fifteenth century. Thatta is mentioned as `Masqat's most important Indian trading partner', and its Hindu merchants, the Bhatias, appear to have been the main participants in the trade between Sind and Arabia [29].

Several historians [5, 30] and Gazetteers have accounted the story of the decline of Thatta from once town of commercial importance to ruins. Edward Thornton describes it in the mid-nineteenth century as “a town formerly very famous, but now much decayed… situated about three miles west of the right or western bank of the Indus” and that its extensive ruins are scattered to ten miles in the south and three miles to the north-west. The population of Thatta in 1699 is estimated to be about 150,000 but after being marred by a plague epidemic, in 1854 the population is estimated to be less than 40,000^29^ and by the beginning of nineteenth century, it was reduced to 20,000 [31]. Pillaged and burned by Portuguese mercenaries^30^ in 1555, Thatta regained some of its prosperity with the arrival of Dutch East India Company between 1652 and 1660, but its revival was short lived as the Indus River silted in the later years of the seventeenth century [32]. It shifted its course further east which led to the abandonment of the city as a seaport [24].

Despite the abandonment of the port functions of Thatta, its Bhatia merchants continued to play an important role in trade, and began using their own ships rather than relying on European ships for trade. Traders were particularly active in the region around Masqat, in modern Oman, and members of Thatta's Bhatia caste established Masqat's first Hindu temple during this period [29]. Both Marcovits and Allen also state that they have extended their activities in the Gulf to new areas, such as the Bahrain islands [24, 29] which is where some of the informants who participated in this research are based today.

## Contemporary food habits of *Thattai Bhatia* community

As far as modern foodways are concerned, the everyday household food strictly follows the religious restrictions. In many households, the ritual of *Bhog Dharanu* is practiced every day, as described by our informant and the author of the book *Panja Khada* discussed in the former sections of the paper. The community still practices the rituals of *Chappanbhog* and *Annkut* during *Goverdhan Puja*. Many unsaid rules are also observed while consuming *Thattai Bhatia* meal like the diner must suck the juices from the *singhi* (drumsticks), chew on the cubes of potato, yam and banana, and leave out the curry leaves, *kokum phool* (wild mangosteen) and *kelay jo chilko* (banana peels) [33]. Unlike other north Indian communities, the Thathai Bhatias begin the lunch with rice, and follow it up with the *roti, phulka and poori* (types of flatbreads). Many non-alcoholic drinks called *sherbet* made up of sandalwood, jasmine and rose are also a popular feature of the Thattai Bhatia meal as the ingredients of *sherbet* are refreshing in nature, they are more suitable for a cuisine developed around deserts [33].

However, the younger generation is quiet flexible in and is evolving in terms of their food choices. My informant stated that the newer generation is less strict in their food choices and do not refrain themselves from trying other cuisines like “Chinese, Italian and Mexican” and also consume alcohol socially as a personal preference.

## Conclusion

It is nearly impossible to study the history of Jaisalmer in Rajasthan without studying the valour stories of the Bhatti clan and the Bhatner Fort [34] which is one of the oldest forts in India. Likewise, the merchants of Sindh and especially the city Thatta as a commercial hub has been a subject of interest to many scholars (Marcovits, Subhramanyam), Thatta being one of the richest cities of the Orient as per the chronicles of Diego de Couto [5]. Similarly, the *Thattai Bhatia*s are a theme of great interest as a Hindu minority diaspora in the Muslim dominated Gulf countries (Jain, Mathew, and Khalid). The three have been prospective research topics in their own capacity. However, it is their culinary identity, “what they eat” made us see all three of them as chronologically dependent events of history shaping the foodways of the community to what it is today. Their everyday food revealed the answers to their similarity yet distinctness from both Sindhi and Rajasthani cuisine. It also brought to light the potential role, faith can play in shaping the cuisine. The paper bared the history of the community by tracing back the salient characteristics of the *Thattai Bhatia* cuisine. Thus, culinary identity proved to be an effective method to study history of any community of which there is little or no documentation of culinary regime. The method may not necessarily always precisely converge at one point but reserves the potential to streamline the course of the research. The study has established the reasoning behind the identification which their ethnic cuisine provides them.

The research revealed that the *Thattai Bhatia* community underwent several migrations and has very small presence but with their exemplary efforts, they have managed to practice and maintain a distinct cuisine undeterred by their migrations. Despite its resemblance with Sindhi and Gujarati cuisine both in terms of ingredients and nomenclature, *Thattai Bhatia* cuisine is unique and discrete in many ways and clearly revolves around their faith. The cuisine provides a spread of pre-planned vegetarian *sattvik* meals and is worthy of marking a presence on the global map. The community is now assiduously working towards recording their history under the name of The Bhatia History Project [35] announced in 2020. Historians from multiple nations will be working on it to converge facts from India, Pakistan and the Persian Gulf. Probably, it is now that they have all the “resources”, which Appadurai [4] referred to in the context of cookbooks, to record and document the history. This research may be a drop in the ocean, but I believe that The Bhatia History Project will generously benefit from it.

**Fig. 2:**
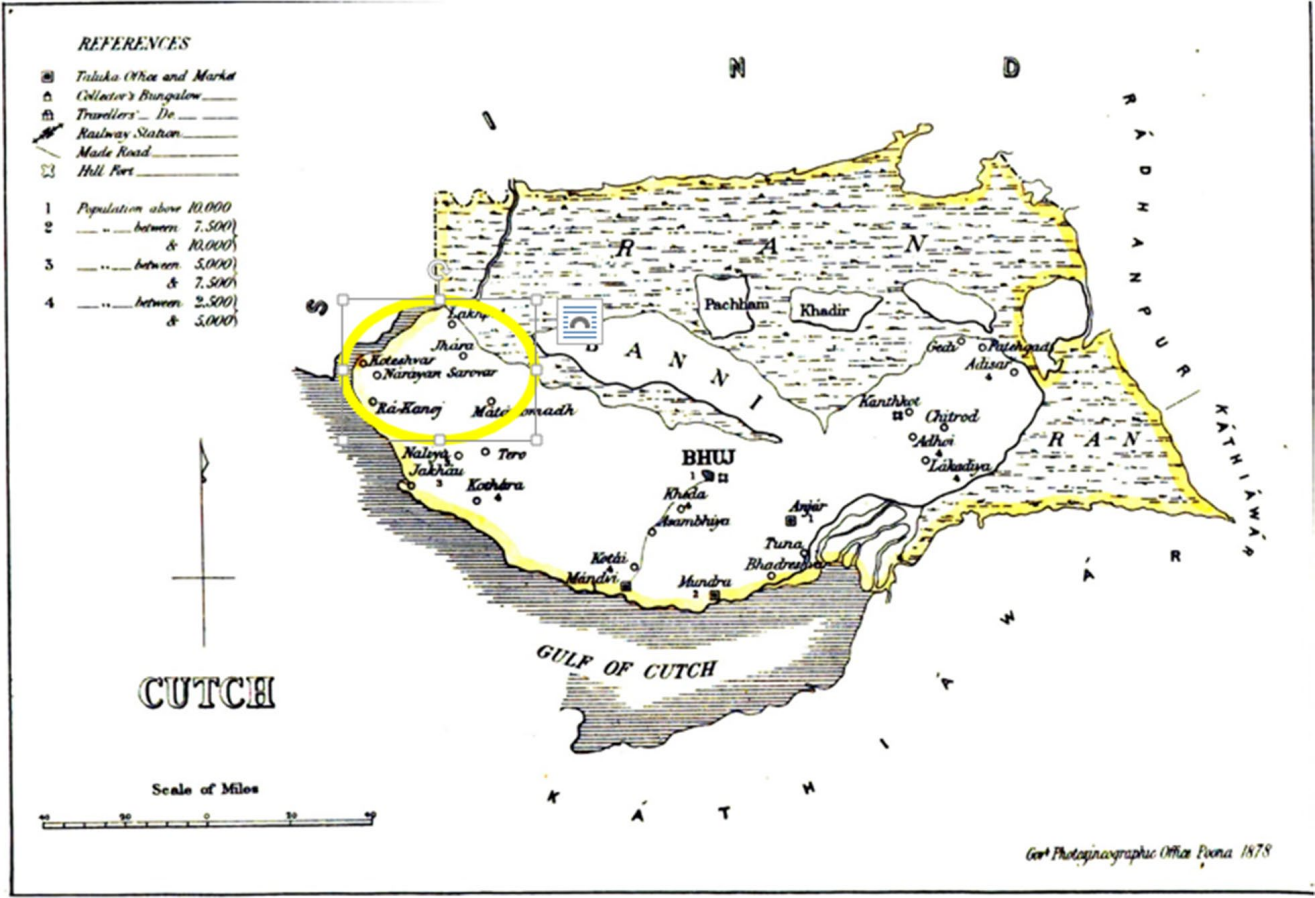
The map depicting the location of Narayan Sarovar in Kutch. *Source*: “Chapter XIII: Places of Interest” in Gazetteer of the Bombay Presidency: Cutch, Palanpur, and Mahi Kantha (Government Central Press 1880)

**Fig. 3:**
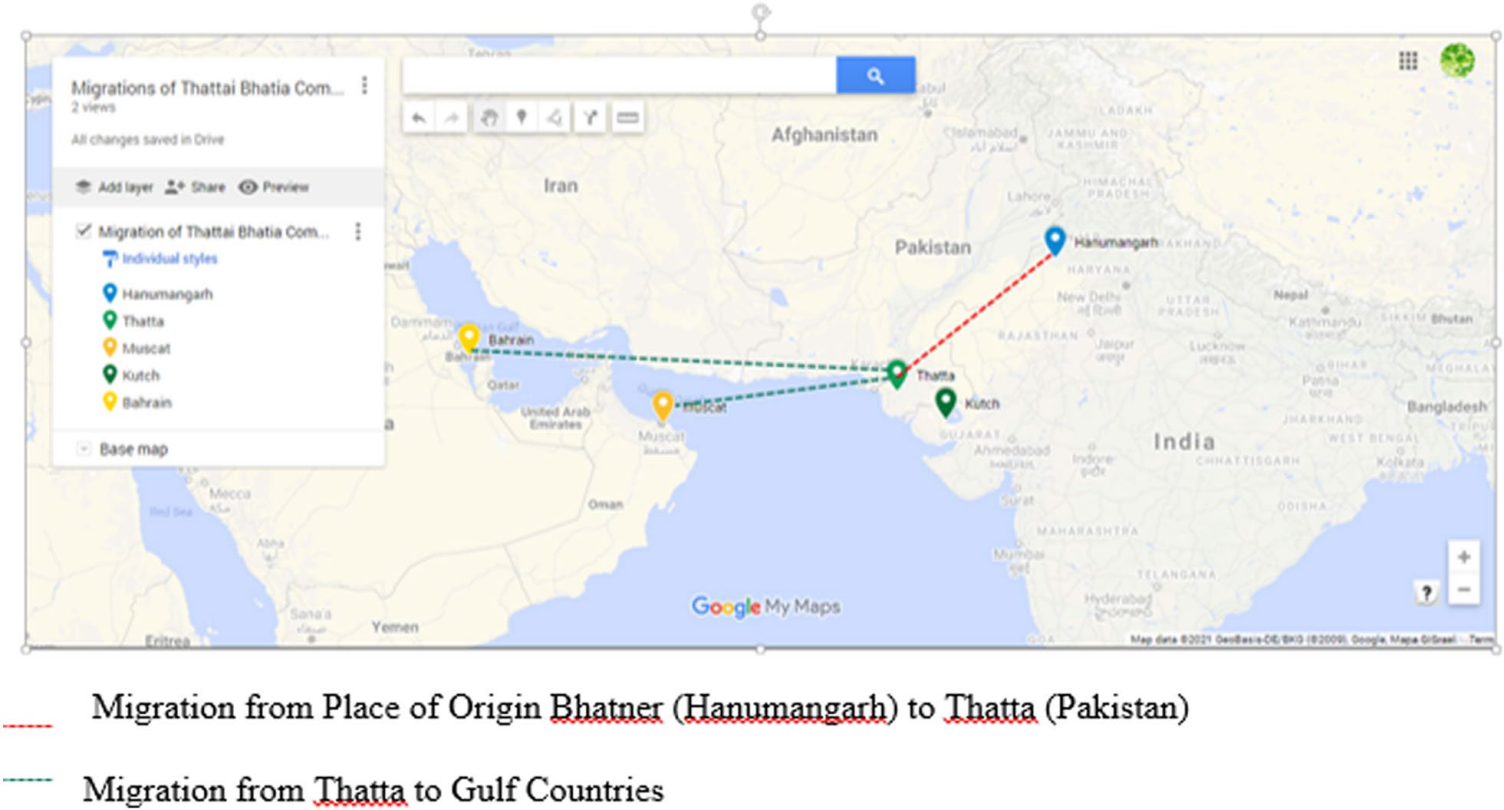
Map depicting important places and migration routes of the Thattai Bhatia community (Map created using Google My Map)

## Data Availability

The data from the interviews are available with the author.

## Footnotes

1 Nala is a character in the epic *Mahabharata*. The legend is that his wife Damyanti was attracted to the smell of the kitchen that clung on to him and masked the of smell fragrances worn by other men during the *swayamwara* (an event in which a women chooses her husband among any men present) and married him when he was a deployed as a cook in the kingdom of Ayodhya. https://devdutt.com/articles/chef-nala/.

2 Tribal people who considered the hills and the forests their abode.

3 Derived from *Tat*- means bank of a river. The Sindhi word for river bank is *Thatto*. Thatta is also locally termed as Nagar Thato or Thatta Nagar.

4 Sindhi Lohanas are further divided into *Amils, Bhaibands, Sahitis and Ladii Lohana* based on their profession, Mark-Anthony Falzon, *Cosmopolitan Connections: The Sindhi Diaspora, 1860–2000* (Brill Academic Publishers 2004), p. 35.

5 Belonging to a place called Aror also known as Arror, Alor or Arorkot is the medieval name of the city of Rohri in modern day Pakistan. The city once served as the capital of Sindh.

6 *Baniya*, derived from Sanskrit word *vaniya* or *vanijya*, meaning finance or commerce. The Hindi word for traders is *baniya*.

7 The study was conducted during Covid-19 travel restrictions; thus, I had to depend on the technology medium to conduct the interviews.

8 The Official Thathai Bhatia Food Website (*Panja Khada)* http://www.panjakhada.com.

9 Gujarat is a state on western coast of India.

10 Rajasthan is the largest state of India in terms of area and is bordered by Gujarat in the southwest.

11 Steamed, sometimes fried dumplings made up of *besan.* The name derives from the word “*mutthi”* meaning fist. The dumpling is shaped by tightly holding the floor in the hand giving it the shape of the fist and is a classic Gujarati snack.

12 Sweetmeat made up of *besan*, *ghee*, sugar and nuts.

13 Fried *besan* snack in tomato gravy, commonly made in Rajasthan.

14 Steamed savory cake made up of *besan* and curd and tempered with curry leaves and mustard seeds.

15 Wheat-flour crumble with nuts, a distinct Rajasthani food item.

16 Gujarati cuisine uses Bengal gram extensively and *vaghar* (tempering, in hot oil and spices) is a common feature in Gujarati food.

17 Both curries made up of potato and lotus stem. Sindhi have a special liking for lotus-stem and lotus seed.

18 Twice fried and spiced potato, yam or taro root.

19 A curry of Bengal gram with vegetables like drumsticks, okra, banana peels, lotus-stem soured with *kokum* (fruit used as souring agent). *Sindhi kari* is an essential item on the menu during wedding ceremonies.

20 A combination of spiced and tempered Bengal gram curry and wheat or refined flour crisps which serves as a breakfast recipe.

21 Several Sindhi dishes are based on garlic only for flavour like *thoom ja vada* meaning a patty made up of garlic greens, something completely absent from Bhatia cuisine.

22 *Palla* or pallo (Tenualosa ilisha) also known as *illish* or *hilsa*. It is a Sindhi delicacy and holds a sacred place in their lives. It was abundantly found in Indus and was locally called *Darya ka phool*, meaning the flower of the river. Jhulelal, the deity of Sindhis is believed to ride on a *palla* (Fig. 2). *Palla* finds no place in the Bhatia cuisine.

23 *Macroli* or what we commonly known as macaroni has found a place in Sindhi cuisine. *Macroli phoolpatasha* (macaroni and dried lotus seed/fox nut) and *macroli patata* (*macaroni and potato curry*) are the dishes of Sindhi cuisine. It is believed that Sindhi got introduced to macaroni during their trade relations with Europeans. However, no account of this is found in the literature.

24 Burton has documented a mythological story which suggests that the *Pokarno*, the priests of the Bhatiya Banyans (*banias*, traders) were cursed by Goddess Parvati for refusing to eat flesh, they had to migrate from Jaisalmer to Sindh, Richard F Burton, *Sindh, and the races that inhabit the valley of the Indus; with notices of the topography and history of the Province* (WH Allen & Co. 1851) 317.

25 www.bhatias.org.

26 The followers of Lord Vishnu, the other contemporary sect being *Shaivas*, the followers of Shiva.

27 Followers of *Pushtimarg,* also known as *Pushti Sampraday or Vallabha Sampraday.*

28 A path of spiritual nourishment, a Vaishnav tradition. The worship the avatar of Lord Vishnu in the form of Lord Krishna in his childhood form known by other names as *Srinathji* or *Maharaj*. One of the most important shrines of SriNathji is in India known as *Nathdwara*.

29 It is also recorded that; some have estimated the population to be below 2000, Thornton (n 29).

30 Traders in Thatta were trading with the Portuguese colonizers in India in the sixteenth century. Refer (Subhramanyam 1991).
